# Establishment of a m^6^A‐Related Molecular Pattern in the Prognosis and Immune Infiltration of Osteosarcoma Using Machine Learning and Experiments

**DOI:** 10.1155/ijog/2000690

**Published:** 2026-02-14

**Authors:** Na He, Xia Chen, Chunyan Zhang

**Affiliations:** ^1^ Department of Orthopedics, The Second Xiangya Hospital, Central South University, Changsha, Hunan, China, csu.edu.cn; ^2^ Hunan Key Laboratory of Tumor Models and Individualized Medicine, The Second Xiangya Hospital, Central South University, Changsha, Hunan, China, csu.edu.cn

**Keywords:** elastic net penalized Cox regression, N6-methyladenosine, nomogram, osteosarcoma, prognosis

## Abstract

**Background:**

To determine the prognosis of osteosarcoma, multiple predictive models have been constructed in recent years. Nevertheless, the model for N6‐methyladenosine (m^6^A)‐related genes, a critical subset of molecular regulators for osteosarcoma, has not been identified.

**Methods:**

Gene expression matrices and clinical data were extracted from the GEO datasets GSE21257 and GSE16091. Randomly selected 70% of samples from GSE21257 were assigned as the training dataset, while the remaining 30% of samples from GSE21257 and all samples from GSE16091 were designated as the internal test and external test datasets, respectively. The predictive model was developed using elastic net–penalized Cox regression. Receiver operating characteristic (ROC) analysis, Kaplan–Meier analysis, and Wilcoxon′s tests were conducted in the training, internal test, and external test datasets to validate its efficacy. Additionally, a clinical nomogram was established for prognostic prediction. The expression of several signature genes was verified in osteosarcoma cell lines and clinical samples. In vitro experiments were performed to elucidate the impact of signature genes on the osteosarcoma phenotype. Immune infiltration analysis and gene set enrichment analysis (GSEA) were further integrated to validate the ability of the risk model to discriminate cancer characteristics.

**Results:**

A total of 110 m^6^A‐related and survival‐significant genes were identified from GSE21257. Among these, 14 genes were ultimately included in the prognostic model for osteosarcoma. ROC analysis showed that the AUC values in the training, internal test, and external test datasets were 0.8304, 0.9091, and 0.7123, respectively. Furthermore, the AUC values for predicting 1‐, 3‐, and 5‐year overall survival were 0.8827, 0.8709, and 0.7664, respectively, with an overall AUC of 0.8275. Under this framework, a clinical nomogram was successfully constructed. Notably, immune infiltration analysis revealed a reduced immune score in the high‐risk group. GSEA demonstrated enrichment of several well‐known malignancy‐related gene sets in the high‐risk group, including E2F target genes, MYC targets, mitotic spindle, and hypoxia‐related pathways, among others.

**Conclusions:**

A prognostic model based on m^6^A‐related genes was developed, which exhibits strong efficacy in predicting the prognosis of osteosarcoma. Additionally, a robust clinical nomogram was generated, providing novel evidence to support clinical decision‐making and personalized treatment.

## 1. Introduction

Osteosarcoma is the most prevalent bone malignancy with an incidence of 1~3 cases per million worldwide annually [1]. Mainly occurring in adolescents, it has become the second lethiferous tumor among young teenagers [2]. With high malignancy, metastasis has been reported in nearly one‐quarter of patients, especially metastasis to the lung [3]. The clinical manifestation of osteosarcoma involves osteodynia, motion limitation, and spontaneous fracture. However, early diagnosis is difficult because of the unobvious inchoate symptoms in most cases. Currently, the standard therapy for osteosarcoma comprises neoadjuvant chemotherapy, chemotherapy, and surgery. Even though great advancements have been achieved in the past decades, the 5‐year survival of osteosarcoma patients is still less than 60% [4]. Novel therapeutic methods have emerged quickly in recent years, such as targeted therapy and immunotherapy. For instance, a negative correlation between PD‐1 and the prognosis of osteosarcoma has been demonstrated. Further, nivolumab, a PD‐1 blocker, has significant efficacy in suppressing osteosarcoma metastasis [5], while other agents like pembrolizumab and avelumab are still undergoing clinical trials [6]. In the meantime, prognostic prediction of osteosarcoma using various parameters has become a topic of attention. Despite the traditional predictive factors such as age, gender, clinical stage, and metastasis, the transcriptional alteration of osteosarcoma tissue or cells has been partially identified as effective predictors [6].

m^6^A is a dynamic and reversible modification in RNAs through adding or removing methyl to the adenosine [7]. Directly, the installation and uninstallation of m^6^A are accomplished by diverse proteins, namely, “writers” and “erasers”, respectively, while “readers” are in charge of recognizing the m^6^A sites [8]. Importantly, m^6^A has been illustrated to participate in the growth [9], migration [10], invasion [11], and chemoresistance [12] of osteosarcoma. Actually, thousands of human mRNAs were illuminated to be modified by m^6^A [13]; nevertheless, their functional implications in osteosarcoma have not been expounded. Therefore, we conducted a prognostic prediction model, aiming to identify a subset of m^6^A‐related genes that are relevant for discriminating among positive and poor prognosis of osteosarcoma patients.

Currently, a high‐precision machine learning algorithm enables the establishment of an optimal clinical predictive model by using only a few variables. Cox regression is a widely used machine learning method to identify outcome‐related factors. However, the traditional Cox regression is not suitable for selecting only a few variables in high‐dimensional datasets [14]. To cope with this drawback, penalized methods such as elastic net penalized Cox proportional hazards regression have been developed, which provide the opportunity to maximize predictive accuracy and reduce statistical concerns like multicollinearity and high variance [15]. Therefore, to build an optimal model using genomic data, elastic net was adopted in this study.

## 2. Materials and Methods

### 2.1. Data Preparation

Datasets GSE21257 and GSE16091 were obtained from the National Center for Biotechnology Information GEO (https://cancergenome.nih.gov/). Seventy percent of the samples in the GSE21257 dataset were applied as training data, while the rest were applied as internal validation data. GSE16091 was treated as the external verification dataset. For GSE21257 and GSE16091, the normalized gene expression matrix was downloaded and annotated according to the annotation file provided by GPL10295 and GPL96 platforms, respectively. Then, 24,998 genes in 53 samples were identified in GSE21257, while 12,546 genes in 34 samples were recognized in GSE16091.

### 2.2. Identification of Survival Significant Genes in GSE21257

Gene expression of the 53 samples in GSE21257 was separated into two groups (high vs. low) by the median value. R package “survival” was employed to discriminate genes that were significantly (*p* < 0.05 by Kaplan–Meier analysis) correlated with overall survival. Further, the m^6^A‐related genes were determined by the GeneCards database (https://www.genecards.org/).

### 2.3. Establishment of Elastic Net Penalized Cox Regression Model

70% samples in GSE21257 were randomly selected as the training dataset. Elastic net penalized Cox regression was conducted to screen the genomic data and single out variables for the predictive model by using the R package “glmnet”. A combination of optimal *λ* (*λ*_mi*n*_ and *λ*_ls*e*_) was applied to fit the elastic net. Further, the Wilcoxon test and ROC were used to test the efficacy of *λ*_min_ and *λ*_lse_ in distinguishing patients between high‐ and low‐risk groups. Finally, a subset of genes was chosen as the model gene.

### 2.4. Cox Proportional Hazard Regression Model

For the enrolled model genes, univariate Cox regression was performed to assess the correlation between gene expression level and prognosis of patients by using the R package “survminer” and “survival.” The HR value and 95% confidence interval were measured and shown in a forest plot. Further, for each involved single gene, a Kaplan–Meier survival curve was drawn via the R package “survival.”

### 2.5. Construction of Polygenic Risk Score Model

To test the potency of model genes in prognostic prediction, a polygenic risk score model was constructed. Patients were divided into two groups (high risk vs. low risk) by the median value of the calculated risk scores. The division was implemented in both the GSE21257 and GSE16091 datasets. Next, risk‐related Kaplan–Meier survival analysis, ROC analysis, and event distribution analysis were employed to confirm the polygenic risk score model.

### 2.6. Nomogram for Prognostic Prediction

To visualize the prognostic efficacy of risk groups differentiated by the risk score model, a nomogram comprising factors of risk group, age, gender, Huvos Grade 2, and metastasis was established by the R package “rms” to predict the 1‐, 3‐, and 5‐year survival probability. Huvos grade is a pathological grading system for cancer that evaluates tumor cell necrosis degree after neoadjuvant chemotherapy (Grade I: ≥ 95% necrosis, Grade II: 50%–94% necrosis, Grade III: 10%–49% necrosis, and Grade IV: < 10% necrosis), which correlates with chemotherapy response and prognosis (lower grades indicate better outcomes) and guides postoperative treatment adjustment. Calibration curves were plotted for 1‐, 3‐, and 5‐year checkpoints to assess consistency between the nomogram‐predicted probability and the observed rate. Further, an interactive nomogram for survival probability prediction was designed using the R package “regplot.”

### 2.7. Immune Infiltration and Gene Set Enrichment Analysis (GSEA)

To further determine the efficiency of this risk model, the immune infiltration and GSEA were adopted in high‐ and low‐risk groups. We applied the CIBERSORT and XCELL algorithms to assess the immune conditions and the R package “clusterProfiler” for GSEA.

### 2.8. Cell Lines

The hFOB1.19, 143B, U2OS, and MG‐63 cell lines were acquired from the Procell Life Science and Technology Co. Ltd. hFOB1.19, MG‐63, and 143B cells were cultured in DMEM culture media completed with 10% fetal bovine serum and 1% penicillin–streptomycin at 37°C and 5% CO_2_. U2OS cells were cultured in McCoy′s 5A containing 10% fetal bovine serum and 1% penicillin–streptomycin at 37°C and 5% CO_2_.

### 2.9. Real‐Time Quantitative PCR (RT‐qPCR)

Total RNAs were extracted using RNA Express Total RNA Kit (M050, NCM Biotech, China). The RevertAid First Strand cDNA Synthesis kit (K1622, Thermo Fisher Scientific, United States) was applied to synthesize cDNA from the total RNA after the removal of genomic DNA. Sequences of primers are shown in Table S1.

### 2.10. Plasmid Construction

To construct the CRISPR/Cas9 knockout vector, sgRNAs targeting specific genes or a nontargeting control sgRNA were cloned into the lentiCRISPRv2 plasmid. The sequences of sgRNAs are presented in Table S1. 143B and U2OS osteosarcoma cells were transduced with lentiviruses that expressed specific sgRNAs and Cas9. After puromycin selection, Western blotting was used to validate the efficacy of CRISPR/Cas9 knockout.

### 2.11. Western Blotting

Cells were lysed with RIPA buffer containing a protease inhibitor and centrifuged for 15 min at 13,500 g. Protein concentration was determined by BCA Protein Kit (Vazyme). Equivalent amounts of protein from different groups were subjected to SDS‐PAGE electrophoresis. The proteins were then transferred onto PVDF membranes and blocked with 5% nonfat milk for 2 h at room temperature. The membranes were incubated with primary antibodies against MCAM (Proteintech, 17564‐1‐AP), NNT (Proteintech, 13442‐2‐AP), SLC7A1 (Proteintech, 14195‐1‐AP), TRAP1 (Proteintech, 10325‐1‐AP), or beta‐tubulin (Proteintech, 66240‐1‐Ig) overnight at 4°C. After washing with TBST three times, the membranes were incubated with secondary antibodies (Mouse: Proteintech, RGAM001; Rabbit: Proteintech, RGAR001). Proteins were visualized by utilizing ECL substrate (Bio‐Rad, United States).

### 2.12. Cell Proliferation Assay

Cells were seeded in a 96‐well plate with a concentration of 2000 cells per well. After treatment for the indicated durations, cell viability was measured using the cell counting kit‐8 (CCK‐8) according to the manufacturer′s instructions.

### 2.13. Clone Formation Assay

Cells were transfected with indicated siRNAs and seeded in six‐well plates at a concentration of 500 cells per well. Cells were then cultured for 14 days to form colonies. At the end of the experiment, cells were fixed with 4% paraformaldehyde and stained with 0.2% crystal violet solution.

### 2.14. Apoptosis Assay

Cells were seeded in a six‐well plate at 150,000 cells/well and cultured overnight. Subsequently, cells were transfected with indicated siRNAs and cultured for 48 h. At the end of the experiment, cells were digested and incubated with Annexin V‐PE and 7‐AAD for 10 min in the dark. Finally, the stained cells were tested using Cytek NL‐CLC within 1 h.

## 3. Results

### 3.1. Construction and Validation of the Elastic Net Penalized Cox Regression Model

**Figure 1:**
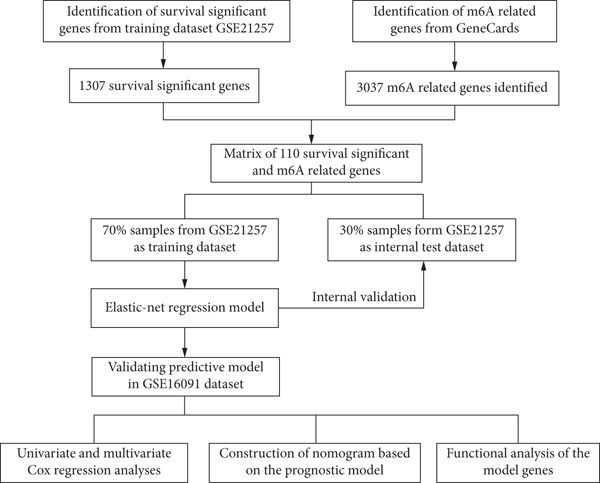
The workflow used to construct and validate the prognostic model.

The detailed workflow of this study is described in Figure 1. Firstly, 1307 survival significant genes were filtered in GSE21257, and 3037 m^6^A‐related genes were identified from GeneCards. The m^6^A‐associated genes mean that they have been certificated to regulate the m^6^A modification or be modulated by m^6^A. After taking the intersection, an expression matrix of 110 genes was inputted to perform elastic net penalized Cox regression. Then, 70% randomly selected samples from GSE21257 were initially enrolled to establish the prognostic model. Two *λ* values (*λ*_min_ = 0.2799, *λ*_lse_ = 0.7096) were obtained to fit the optimal prognostic model (Figure 2a,b). Next, two elastic net Cox regression models were reconstructed based on the *λ*_min_ and *λ*_lse_ values, respectively. The efficacy of the two models was evaluated via assessing the risk score distribution. Consequently, the prognostic model created according to *λ*_min_ showed greater potency than *λ*_lse_ according to the Wilcoxon test (*p* = 0.00036) (Figure 2c). Thus, this model was applied in the subsequent analysis. The model genes were comprised of *CASP8AP2*, *DNMT1*, *GTF2F1*, *KCNK7*, *KRT14*, *LATS1*, *MCAM*, *NAV2*, *NIN*, *NNT*, *NR4A3*, *PPOX*, *SLC7A1*, and *TRAP1* (Table S2).

Figure 2Construction of the 14 m^6^A‐related genes predictive model. (a) Two optimal lambda (l) values (*λ*_mi*n*_ and *λ*_ls*e*_) were estimated according to the vertical lines with a minimizing mean‐square error. (b) Lasso coefficient profiles of the 110 m^6^A‐related and survival significant genes. (c) ROC curves of the prognostic model based on *λ*_mi*n*_ in the training, internal test, and external test datasets. (d–f) The scatter plot of survival status of patients with osteosarcoma using the Wilcoxon test.

**Figure:**
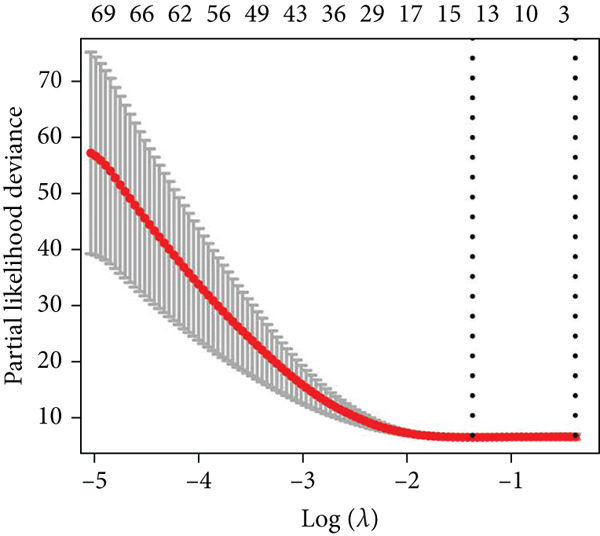
(a)

**Figure:**
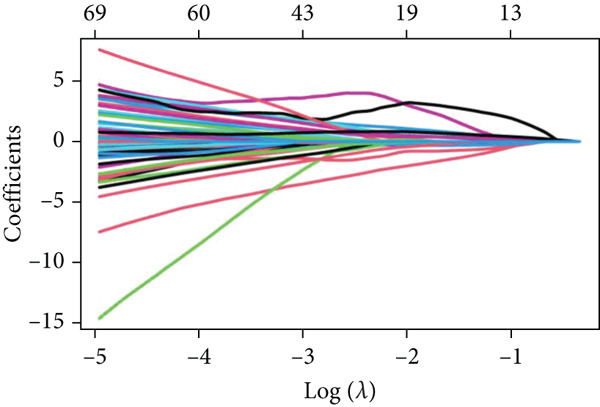
(b)

**Figure:**
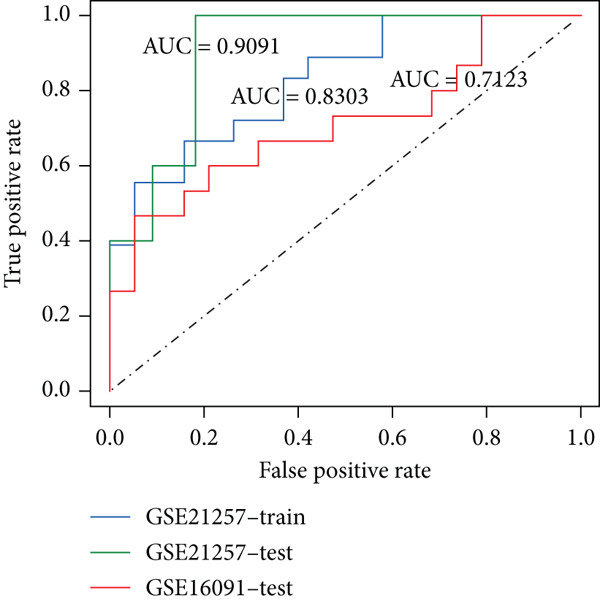
(c)

**Figure:**
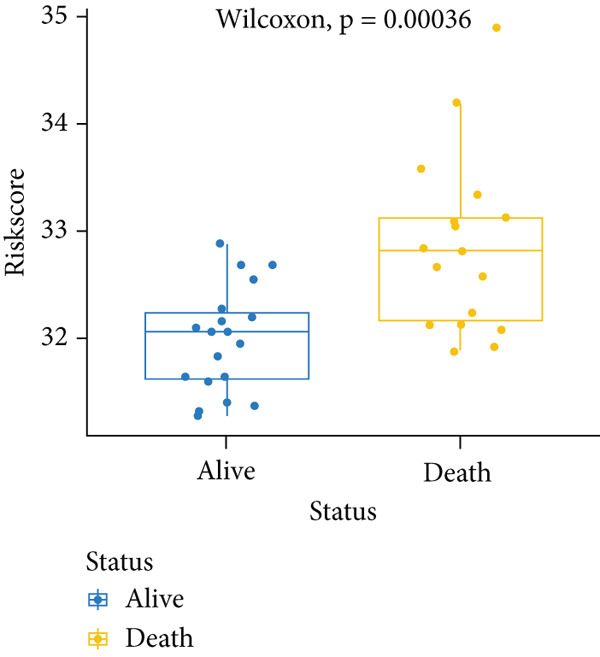
(d)

**Figure:**
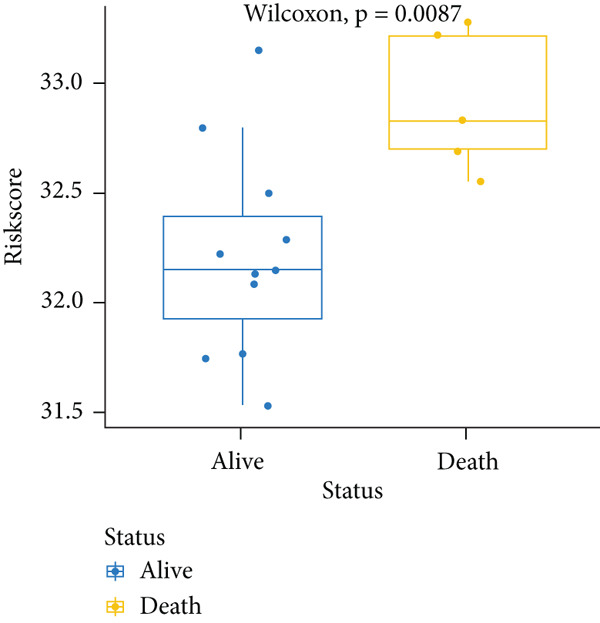
(e)

**Figure:**
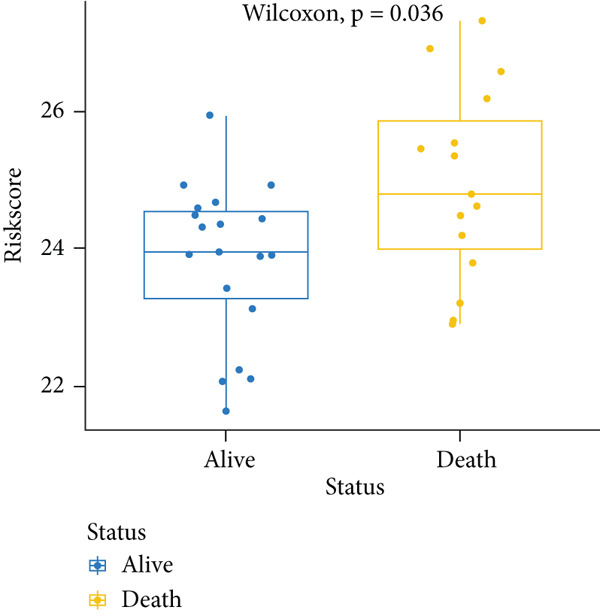
(f)

To notarize the efficacy of this model, internal validation was conducted in the left 30% samples of GSE21257, while external assessment was done in the GSE16091 dataset containing 34 samples. Results revealed that this model showed great prognostic efficacy in both the internal (Wilcoxon′s test, *p* = 0.0087) and external (Wilcoxon′s test, 0.036) validation datasets (Figure 2d,e). Moreover, ROC analysis demonstrated that AUC values in the training, internal test, and external test datasets were 0.8304, 0.9091, and 0.7123, respectively (Figure 2f).

### 3.2. Certification of Signature Genes

We performed Cox regression analyses to figure out the prognostic capability of the model genes independently. Results indicated that in the univariate Cox regression, the *p* value of most genes was less than 0.05 (Figure 3a). Among them, *MCAM*, *NNT*, *SLC7A1*, and *TRAP1* were the risk factors for osteosarcoma, while *KCNK7* was the protective factor. To validate the role of these genes in osteosarcoma, we measured the expression of *MCAM*, *NNT*, *SLC7A1*, and *TRAP1* in osteosarcoma cells and tissues. The results demonstrated that they were overexpressed in osteosarcoma cells compared to the normal counterparts (Figures 3b, 3c, 3d, and 3e). Thus, these signature genes may play critical roles in driving tumor progression.

Figure 3Validation for the prognostic value of the model genes. (a) Univariate Cox regression analysis for the 14 model genes. (b–e) hFOB1.19, 143B, U2OS, and MG63 cells were measured for the mRNA expression of the indicated genes.

**Figure:**
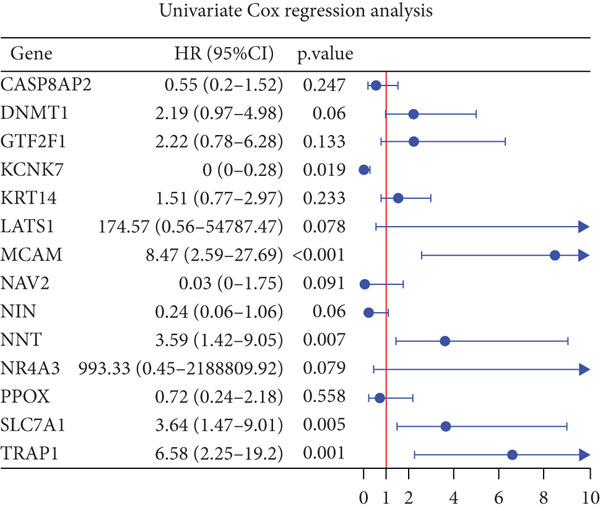
(a)

**Figure:**
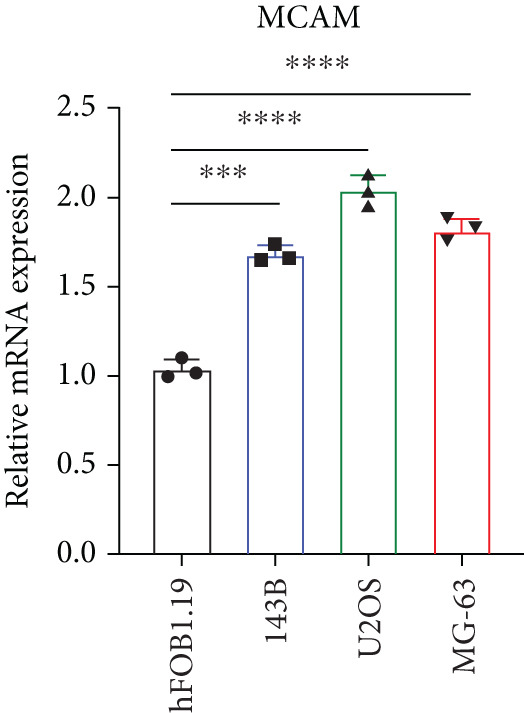
(b)

**Figure:**
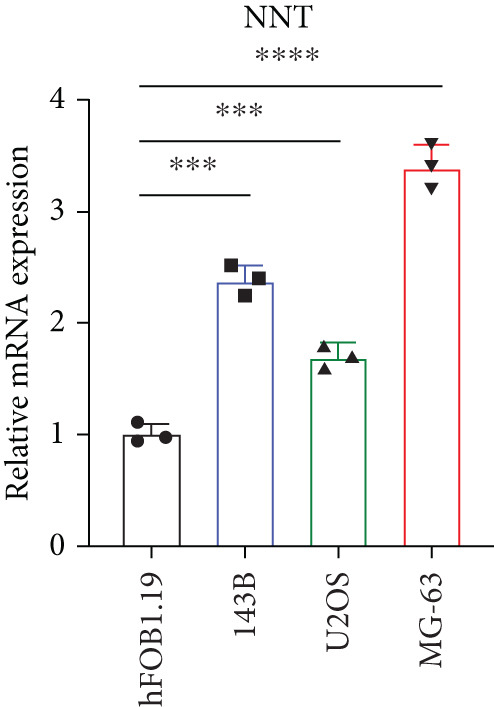
(c)

**Figure:**
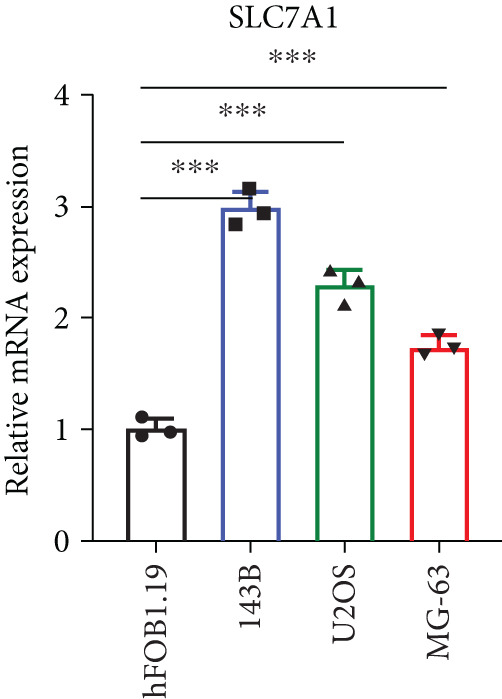
(d)

**Figure:**
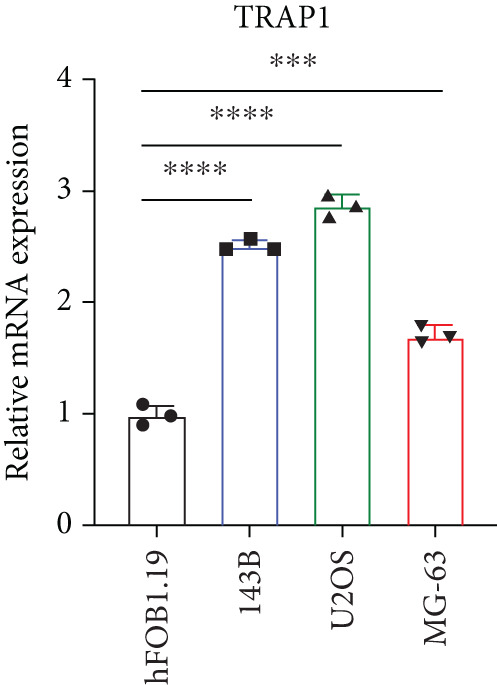
(e)

### 3.3. Establishment and Corroboration of the Polygenic Risk Score Model

Risk scores were calculated based on the elastic net penalized Cox regression model. Patients in the GSE21257 dataset were delaminated into low‐ and high‐risk groups using the median risk score as a cut‐off point (Figure 4a). As presented in Figure 4b, survival was more frequent in patients with a low‐risk score. Besides, time‐dependent ROC analysis illustrated that the overall, 1‐year, 3‐year, and 5‐year prognostic value of the model is satisfactory, with the AUC values of 0.8275, 0.8827, 0.8709, and 0.7664, respectively (Figure 4c). The Kaplan–Meier analysis has illuminated that patients with a high‐risk score tended to have poor prognosis (Figure 4d). Similarly, samples in GSE16091 were classified into high‐risk and low‐risk group according to their risk scores. Likewise, patients with high‐risk scores had a high probability of poor survival (Figure 4e). In the external validation dataset GSE16091, Kaplan–Meier analysis showed that high‐risk patients tended to have poorer overall survival, though the *p* value (0.068) was close to the conventional statistical significance threshold (*p* < 0.05) but did not formally reach it (Figure 4f). This result may be attributed to two factors: first, the relatively small cohort size of GSE16091 (*n* = 34), which inherently reduces statistical power for detecting survival differences; second, the technical heterogeneity between datasets—GSE16091 was annotated using the GPL96 platform, distinct from the GPL10295 platform used for GSE21257 (the training and internal validation dataset). Notably, the survival stratification trend (poorer prognosis in the high‐risk group) in GSE16091 remained consistent with that observed in the training (*p* < 0.0001) and internal validation (*p* = 0.0087) subsets of GSE21257, supporting the risk model′s consistent discriminative ability for osteosarcoma prognosis.

Figure 4Validation for prognostic value of the 14 m^6^A‐related genes model. (a) Scatter plot displaying the risk score of each patient in the GSE21257 dataset. Patients were divided into two groups according to the median value of the risk score. (b) The survival status distribution of patients with different risk scores in GSE21257. (c) Time‐dependent ROC curves validating the prognostic value of the polygenic model for overall, 1‐year, 3‐year, and 5‐year survival. (d) Kaplan–Meier survival curves showing the difference in overall survival between high‐ and low‐risk patients in GSE21257 (log‐rank test, *p* < 0.0001). (e) The survival status distribution of patients with different risk scores in GSE16091. (f) Kaplan–Meier survival curves showing the difference in overall survival between high‐ and low‐risk patients in GSE16091 (log‐rank test, *p* = 0.068).

**Figure:**
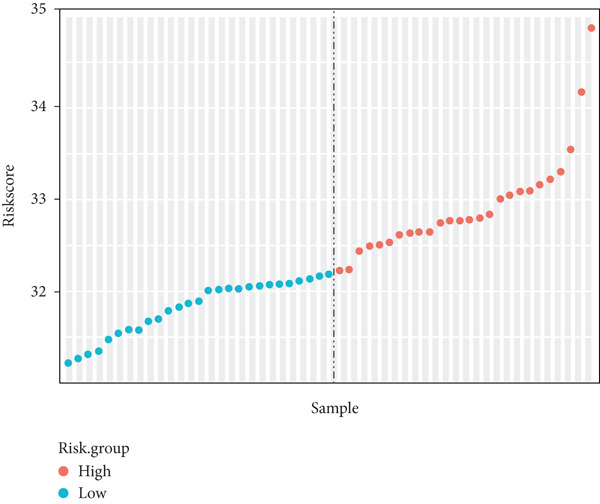
(a)

**Figure:**
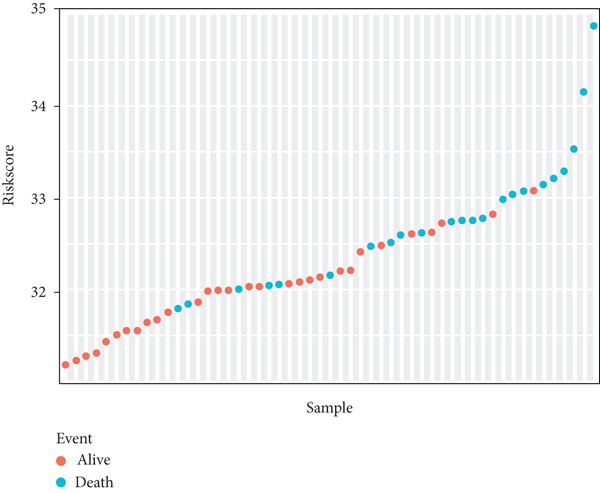
(b)

**Figure:**
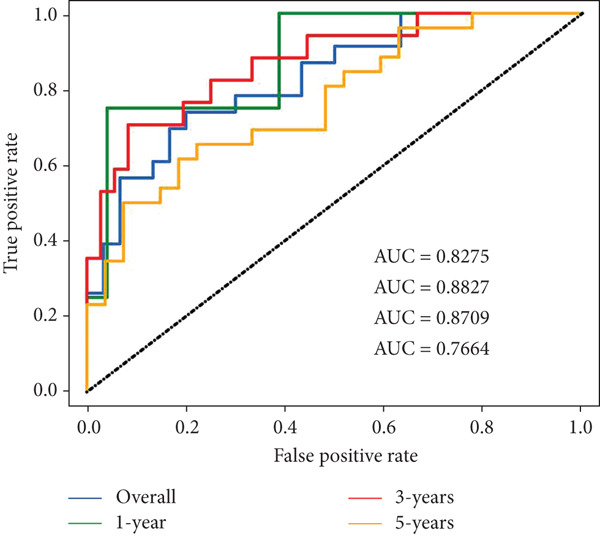
(c)

**Figure:**
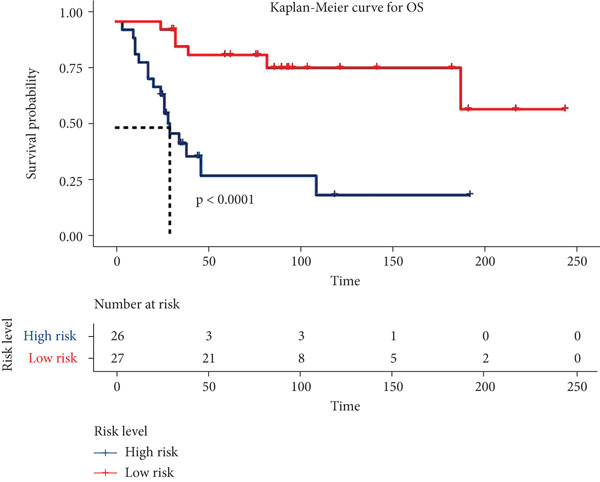
(d)

**Figure:**
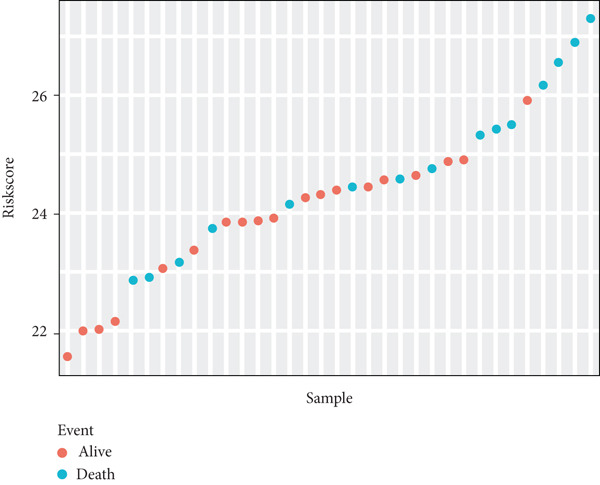
(e)

**Figure:**
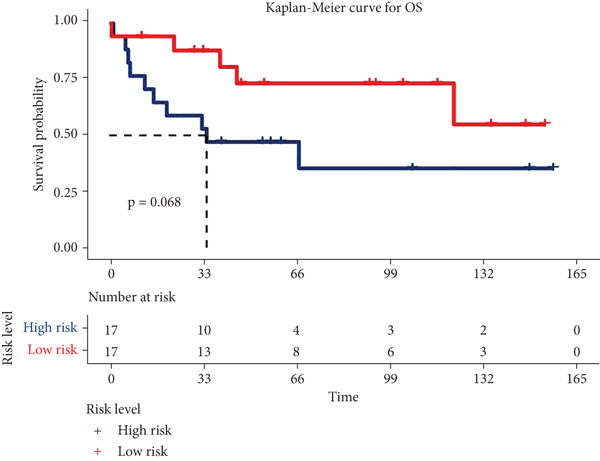
(f)

### 3.4. Construction of Nomogram Based on the Risk Level of Patients

A nomogram containing the variables of age, gender, Huvos Grade 2, risk level, and metastasis was constructed to predict the 1‐, 3‐, and 5‐year survival probability for osteosarcoma patients (Figure 5a). Calibration analysis revealed that the predictive nomogram performed well in survival prediction (Figures 5b, 5c, and 5d). Moreover, to attest to the efficacy of risk level in the nomogram, we assessed the survival of patient GSM531300 in the nomogram presenting or absenting the risk level (Figure 5e,f). This patient lived more than 123 months after diagnosis. However, the nomogram without risk level implied that the probability of death at 60 months was 0.167, which was much higher than that in the nomogram containing risk level (60 months death probability: 0.0755). Therefore, our predictive nomogram enrolling the risk level could forecast the prognosis with higher accuracy.

Figure 5Nomogram predicting the 1‐, 3‐, and 5‐year survival probability of overall survival in patients with osteosarcoma. (a) Nomogram enrolling the variables of age, gender, Huvos Grade 2, metastasis, and risk level. (b–d) Calibration plot for measuring the efficacy of the nomogram in survival prediction. (e, f) Predicting the survival of specific patient GSM531300 by using the nomogram plot enrolling risk level as a variable or not enrolling risk level.

**Figure:**
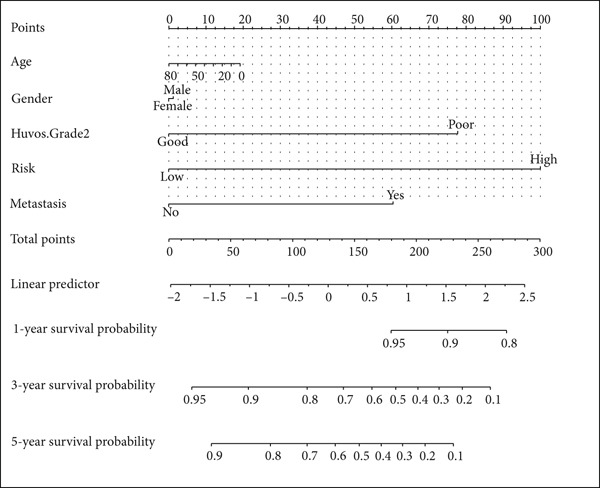
(a)

**Figure:**
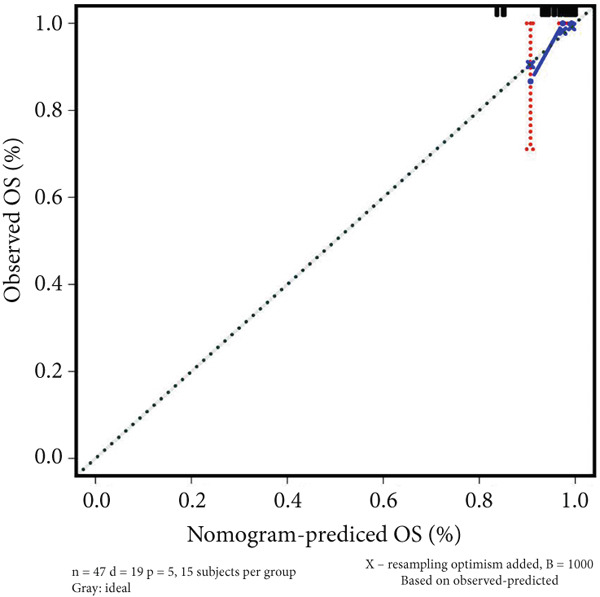
(b)

**Figure:**
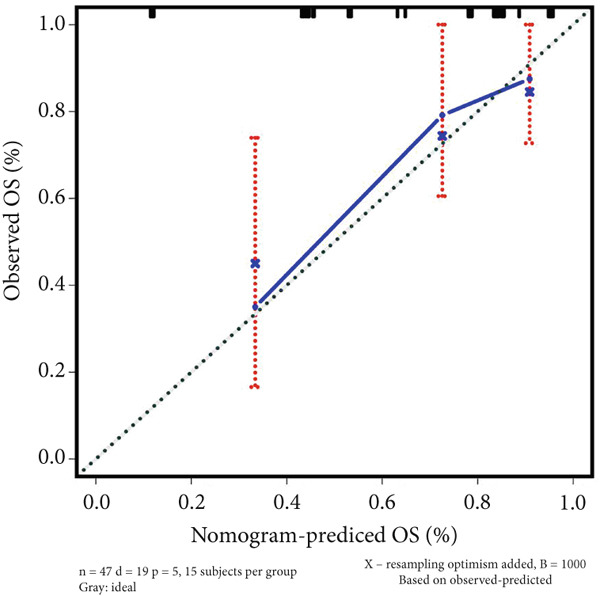
(c)

**Figure:**
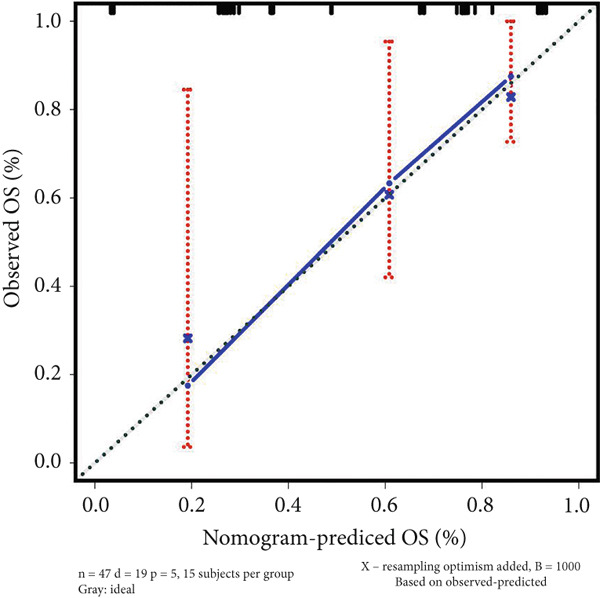
(d)

**Figure:**
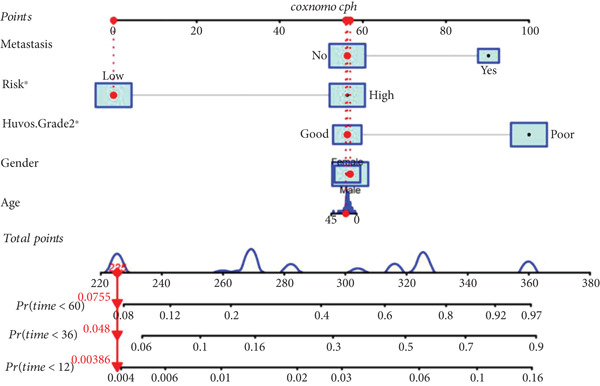
(e)

**Figure:**
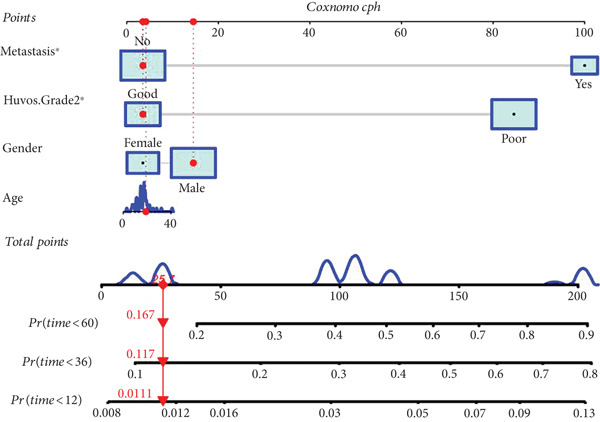
(f)

### 3.5. Immune Infiltration and GSEA in Different Risk Groups

To integrate the potential of this risk model in discriminating cancer behaviors such as immune infiltration and pathway activation, we compared the immune cell composition and immune score between high‐ and low‐risk groups. Results revealed a significant increase in macrophage M0 and a reduction in immune score in the high‐risk group among the GSE21257 dataset (Figure 6a), while macrophage M2 and immune score were both decreased for the high‐risk group in the GSE16091 dataset (Figure 6b). Meanwhile, with the threshold of adjusted *p* < 0.05, FDR *q* < 0.25, and |NES| ≥ 1, GSEA has shown enrichment and activation of several tumor promotion–related pathways such as E2F target genes, MYC targets, mitotic spindle, and hypoxia. Pathways for tumor suppression, including the interferon‐*γ* response and apoptosis, were observably inhibited (Figure 6c,d). These findings suggest the high efficiency of this risk model in differentiating the tumor microenvironment and pathways.

Figure 6Immune infiltration analysis and GSEA. (a, b) Immune infiltration conditions in GSE21257 and GSE16091 datasets. (1) B cell naive, (2) B cell memory, (3) B cell plasma, (4) T cell CD8+, (5) T cell CD4+ naive, (6) T cell CD4+ memory resting, (7) T cell CD4+ memory activated, (8) T cell follicular helper, (9) T cell regulatory (Tregs), (10) T cell gamma delta, (11) NK cell resting, (12) NK cell activated, (13) monocyte, (14) macrophage M0, (15) macrophage M1, (16) macrophage M2, (17) myeloid dendritic cell resting, (18) myeloid dendritic cell activated, (19) mast cell activated, (20) mast cell resting, (21) eosinophil, (22) neutrophil, (23) immune score, (24) stroma score, and (25) microenvironment score. (c, d) GSEA for GSE21257 and GSE16091 datasets.

**Figure:**
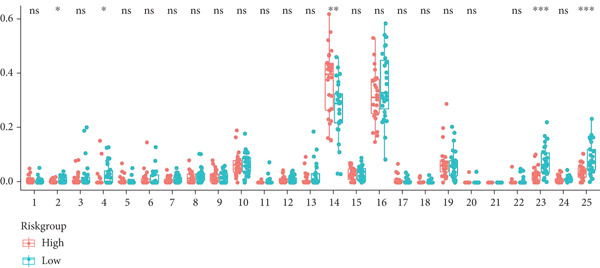
(a)

**Figure:**
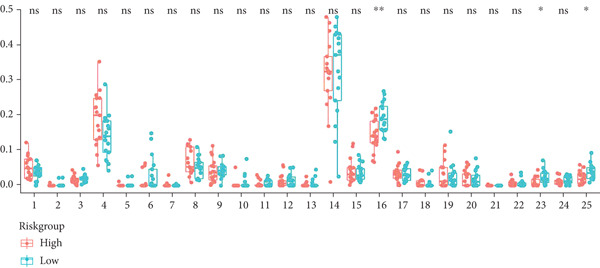
(b)

**Figure:**
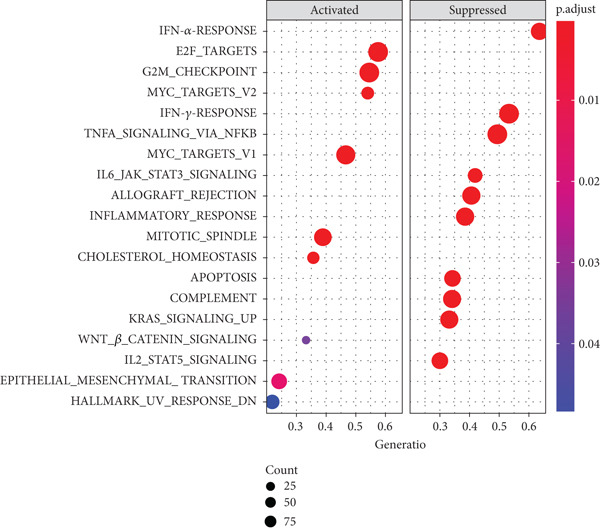
(c)

**Figure:**
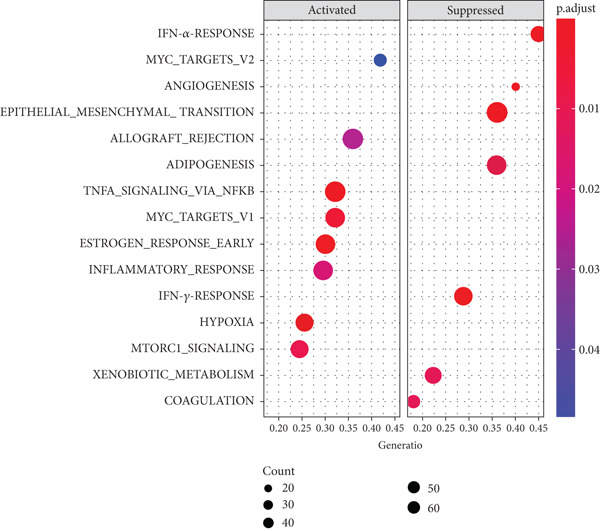
(d)

### 3.6. Effects of TRAP1 on Osteosarcoma Cells

Since TRAP1 was overexpressed in osteosarcoma and associated with poor prognosis, we further validated the phenotypes of TRAP1 knockout. The results suggested that knockdown of TRAP1 significantly suppressed the proliferation of osteosarcoma cells (Figures 7a, 7b, 7c, 7d, and 7e and Figure S1), as indicated by CCK‐8 and colony formation assays. Moreover, knockdown of TRAP1 increased the apoptosis of osteosarcoma cells (Figure 7f,g). Therefore, TRAP1 plays a critical role in driving the progression of osteosarcoma.

Figure 7The effects of TRAP1 on osteosarcoma. (a) 143B and U2OS cells were infected with the indicated lentivirus and measured for TRAP1 protein expression. (b, c) 143B and U2OS cells were infected with the indicated sgNC, sgTRAP1#1, or sgTRAP1#2. After puromycin selection, cell viability was analyzed using a CCK‐8 assay kit at different time points. (d, e) 143B and U2OS cells were infected with the indicated sgNC, sgTRAP1#1, or sgTRAP1#2. After puromycin selection, cells were seeded in a six‐well plate at a concentration of 600 cells per well for the colony formation assay. (f, g) 143B and U2OS cells were infected with the indicated sgNC, sgTRAP1#1, or sgTRAP1#2. After puromycin selection, cells were used for apoptosis analysis.

**Figure:**
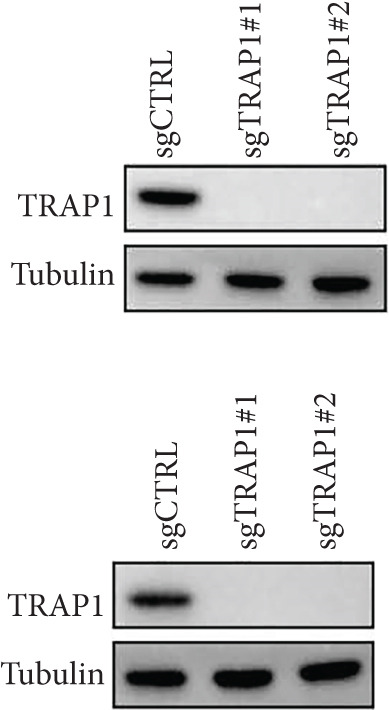
(a)

**Figure:**
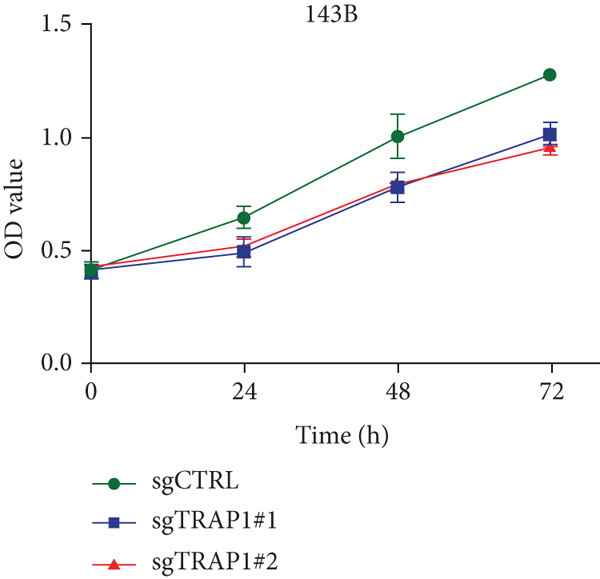
(b)

**Figure:**
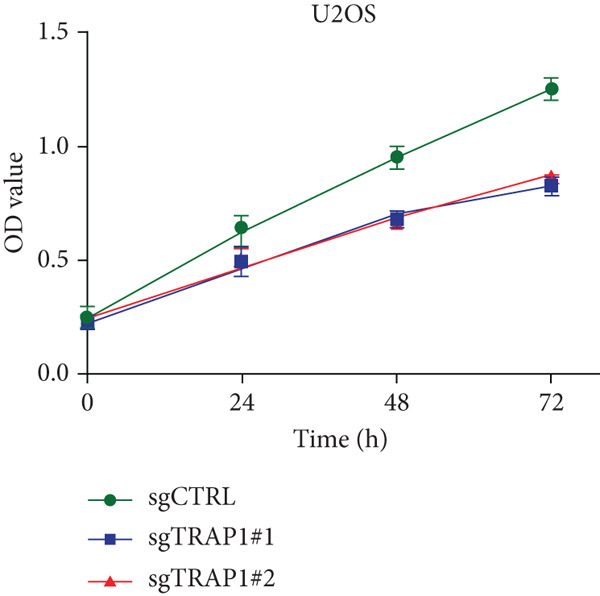
(c)

**Figure:**
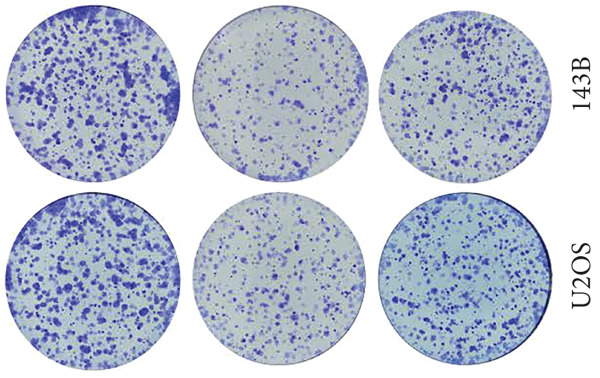
(d)

**Figure:**
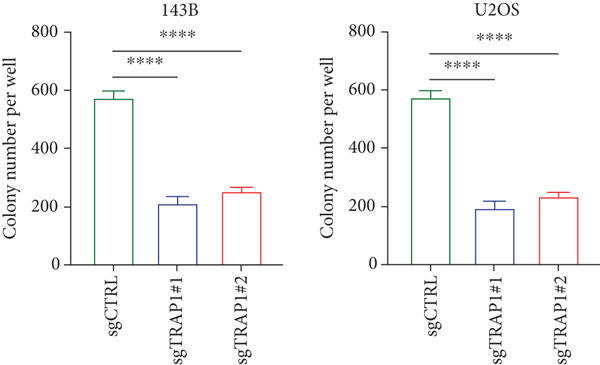
(e)

**Figure:**
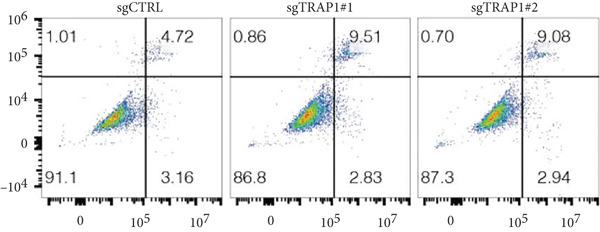
(f)

**Figure:**
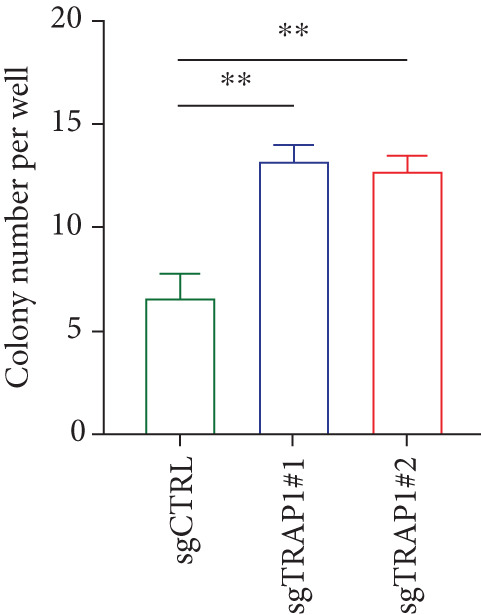
(g)

## 4. Discussions

With high malignancy, osteosarcoma is of great mortality, even though multiple remedies have been implied. Nowadays, the individualized treatment has provided us with new approaches for cancer management [16]. However, the prognostic prediction of patients remains elusive, which greatly impedes the development of customized therapy and decision‐making. In fact, traditional clinical parameters such as age, gender, race, clinical stage, and metastasis have been applied to construct the predictive model for carcinoma. As an example, Wang et al. have developed a nomogram prognostic model involving the factors of age, sex, race, ethnicity, TNM stage, treatment type, and so on for small cell lung cancer [17]. Similarly, by using data from the surveillance, epidemiology, and end results database, He et al. established a practical nomogram for prognosticating cancer‐specific survival in patients with osteosarcoma as a secondary malignancy [18]. In this setting, accumulating studies have also attempted to discriminate the subgroups of osteosarcoma by genomic changes. For instance, a prognostic classifier based on ferroptosis and immune‐related genes has been built to stratify the prognosis of hepatocellular carcinoma patients [19]. With these profiles, it has great potential to develop a prognostic model and enhance personalized therapy for osteosarcoma.

In this study, we have generated a 14 m^6^A‐related genes prognostic for the prediction of prognosis and immune infiltration of osteosarcoma using an elastic net Cox regression model. The model genes consisted of *CASP8AP2*, *DNMT1*, *GTF2F1*, *KCNK7*, *KRT14*, *LATS1*, *MCAM*, *NAV2*, *NIN*, *NNT*, *NR4A3*, *PPOX*, *SLC7A1*, and *TRAP1*. Further, a risk score–based polygenetic model and predictive nomogram were developed to fulfill the clinical application of this model. ROC analysis has validated the efficacy of this model accordingly. The AUC values for overall, 1‐year, 3‐year, and 5‐year survival prediction were 0.8275, 0.8827, 0.8709, and 0.7664, respectively, in GSE21257. The nomogram also proposed great prognostic efficacy of the risk level. The nomogram containing the variable of risk level outperformed that not including the risk level. Therefore, this polygenetic model is efficient in prognostic prediction. Findings of immune infiltration analysis and GSEA also demonstrated the high efficiency of this model in discriminating high‐ and low‐risk groups. Immune scores in the two datasets were decreased in the high‐risk group, possibly due to the low immunogenicity in the microenvironment of high malignant osteosarcoma [20]. Further, the tumor promotion pathways were activated, while the suppression pathways were restrained. Among these pathways, the E2F target genes have shown great activity in tumor progression, which was related to DNA replication, cell cycle progression, DNA damage repair, apoptosis, and differentiation [21]. MYC was a well‐known tumor promoter in various human cancers [22].

Considering that our elastic net penalized Cox regression model performed well in prognostic prediction, these model genes may function in osteosarcoma weightily, though seldom have studies focused on them. Among the 14 model genes, *NR4A3* has the highest coefficient, followed by *NAV2*, *LATS1*, *KCNK7*, *GTF2F1*, and *TRAP1*. *NR4A3* is a multifunctional molecule in cancers engaging in cancer cell growth [23], apoptosis regulation targeted by *p53* [24], and cell differentiation and proliferation [25]. Notably, the EWSR1‐NR4A3 variant fusions were a crucial hallmark for chondrosarcoma [26]. *NAV2*, also known as *HELAD1*, *POMFIL2*, *RAINB1*, *STEERIN2*, and *UNC53H2*, was downregulated in osteosarcoma with poor prognosis. It belongs to the neuron navigator family and is highly conserved in vertebrates [27]. Functioning in cell migration, cytoskeletal remodeling, and microtubule dynamics, it was able to modulate the invasion and migration of cancer cells [28]. *LATS1* was another critical regulator in cancers, which was a core component of the Hippo pathway [29]. Moroishi et al. have elucidated that depletion of Hippo pathway kinase LATS1/2 in three different murine syngeneic tumor models suppressed tumor progression, and that the *LATS1/2* knockdown enhanced immunogenicity for tumor vaccine [30]. Thus, the interference of LATS1/2 may offer a novel method to increase the efficacy of immunotherapy. In contrast to *LATS1*, *KCNK7* protein was expressed at low expression in poor prognosis osteosarcoma. It belonged to the inward rectifier subfamily of K_2P_ channels and was linked to the effect of anesthetics [31]. Li et al. have suggested that *KCNK7* may have predictive capacity in esophageal squamous cell carcinoma [32]. *GTF2F1* is Subunit 1 for the general transcription factor *TFIIF*, which binds to RNA polymerase II and recruits it to a promoter [33]. However, there is currently no study toward the mechanism of *GTF2F1* in the pathological process. Further, *TRAP1* is a widely explored HSP90 molecular chaperone that was first identified by Song et al. in 1994 [34]. Among its versatile functions, *TRAP1* is an indispensable regulator in carcinogenesis, regulating cancer cell metabolism [35], adjusting cancer autophagy [36], and modulating mitochondrial dynamics and cancer metastasis [37]. Therefore, *TRAP1* has been deemed a potential target for oncotherapy [38].

Despite the functional implications in the recent literature, there is still a need to explore the m^6^A‐based mechanisms of these model genes. As aforementioned, they have been identified as the m^6^A‐related genes in the database [39]. Additionally, the GO has identified that most of the model genes are associated with protein‐binding and nucleotide‐binding, which indicates the high probability of their role in m^6^A modification. With further investigation, we may uncover the m^6^A‐associated functional network of the 14 model genes, which enables the finding of novel prognostic markers for osteosarcoma.

## 5. Conclusions

A polygenic prognostic model for osteosarcoma has been established by using the elastic net penalized Cox regression. This model showed great efficacy in the prediction of survival for osteosarcoma patients. In addition, the predictive model‐based clinical nomogram has been built, which may be applied in clinical practice and provide us with novel directions for individualized management of osteosarcoma.

## Conflicts of Interest

The authors declare no conflicts of interest.

## Author Contributions

C.Z. and N.H.: conceptualization, methodology, formal analysis, investigation, visualization, data curation, and writing—original draft. X.C.: conceptualization, methodology, writing—review and editing, and supervision.

## Funding

This study was funded by the Health Commission Scientific Research Program of Hunan Province (D202314027175), the Clinical Research Center for Intravenous Therapy in Hunan Province (2023SK4026), the Hunan Provincial Natural Science Foundation of China (2023JJ30746), and the Changsha Natural Science Foundation of China (kq2208316).

## Supporting information


**Supporting Information** Additional supporting information can be found online in the Supporting Information section. Table S1: The sequences of primers and sgRNAs. Table S2: Elastic net coefficients of the 14 m^6^A‐related prognostic genes. Figure S1: Uncropped full‐length membrane of WB in Figure 7a.

## Data Availability

The datasets generated during and/or analyzed during the current study are available from the corresponding authors upon reasonable request.
