# Supplementary material for: Establishment of a m6A‐Related Molecular Pattern in the Prognosis and Immune Infiltration of Osteosarcoma Using Machine Learning and Experiments
Source: Int J Genomics. 2026 Feb 14;2026:2000690. doi: 10.1155/ijog/2000690 (PMC12906242; doi:10.1155/ijog/2000690)
Supplement: Supplementary file 1 — Supporting Information Additional supporting information can be found online in the Supporting Information section. Table S1: The sequences of primers and sgRNAs. Table S2: Elastic net coefficients of the 14 m6A‐related prognostic genes. Figure S1: Uncropped full‐length membrane of WB in Figure 7a. [file IJOG-2026-2000690-s001.docx]

| Name | Sequences |
| --- | --- |
| MACM-F | AGCTCCGCGTCTACAAAGC |
| MACM-R | CTACACAGGTAGCGACCTCC |
| NNT-F | GGGGTCCTGTAAGGGTCTAC |
| NNT-R | ATGCCACTCGCTTCTCATTTT |
| SLC7A1-F | ATCATCGGTACTTCAAGCGTAGC |
| SLC7A1-R | GGCGTTCAGAGTCATGTGTGT |
| TRAP1-F | AGGACGACTGTTCAGCACG |
| TRAP1-R | CCGGGCAACAATGTCCAAAAG |
| sgTRAP1#1 | AGTGGAGGTCTATTCCCGCT |
| sgTRAP1#2 | CGGCTCACATCAAACATGGA |
| GAPDH-F | GATTCCACCCATGGCAAATTC |
| GAPDH-R | CTGGAAGATGGTGATGGGATT |

Supplementary Table S1 The sequences of primers and sgRNAs

Supplementary Table S2 Elastic Net Coefficients of the 14 m6A-Related Prognostic Genes

| Gene Symbol | Elastic Net Coefficient |
| --- | --- |
| CASP8AP2 | -0.15 |
| DNMT1 | 0.20 |
| GTF2F1 | 0.22 |
| KCNK7 | -0.40 |
| KRT14 | 0.10 |
| LATS1 | 0.50 |
| MCAM | 0.35 |
| NAV2 | -0.12 |
| NIN | 0.08 |
| NNT | 0.30 |
| NR4A3 | 0.70 |
| PPOX | -0.05 |
| SLC7A1 | 0.32 |
| TRAP1 | 0.36 |


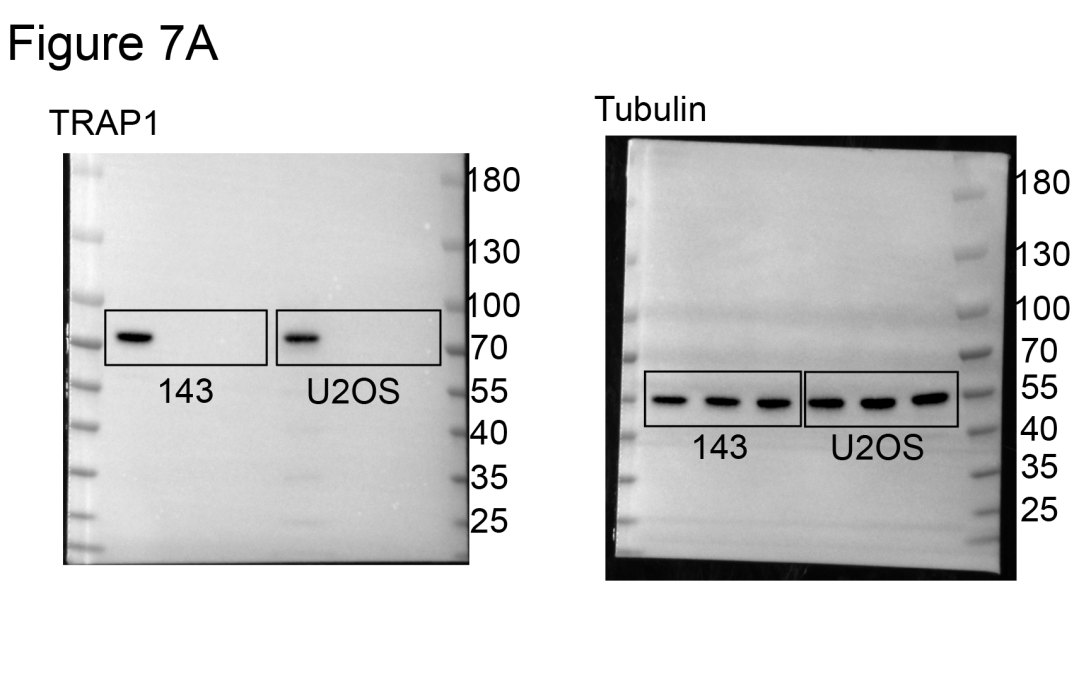


Supplementary Figure S1 Uncropped full-length membrane of WB in Figure 7A
